# Multicenter randomized control study of the efficacy of SO clip in colorectal endoscopic submucosal dissection (ESD). (SO clip study in colorectal ESD): Randomized controlled trial

**DOI:** 10.1097/MD.0000000000033756

**Published:** 2023-05-12

**Authors:** Shinya Taki, Mikitaka Iguchi, Kazuhiro Fukatsu, Toshio Shimokawa, Ikuharu Kinoshita, Ogata Syunsuke, Takao Maekita, Jun Kinoshita, Masaki Takao, Masayuki Kitano

**Affiliations:** a Second Department of Internal Medicine, Wakayama Medical University, Wakayama, Japan; b Department of Gastroenterology, Wakayama Rousai Hospital, Wakayama, Japan; c Clinical Support Center, Wakayama Medical University, Wakayama, Japan; d Department of Gastroenterology, National Hospital Organization Minami Wakayama Medical Center, Wakayama, Japan; e Department of Endoscopy, Kishiwada Tokushukai Hospital, Kishiwada, Japan.

**Keywords:** ESD, colorectal epithelial neoplasm, S-O clip, traction

## Abstract

Endoscopic submucosal dissection (ESD) allows the en bloc resection of colorectal epithelial tumors regardless of size. Although ESD is minimally invasive and yields favorable outcomes, it is technically difficult and requires a long procedure time. In addition, colorectal ESD is associated with a particularly high risk of complications, due to the thin bowel wall, bowel flexion, and peristalsis.

Direct visualization of the submucosal layer by traction of the lesion after mucosal dissection would make ESD performance easier. S-O clips traction lesions toward the lumen, facilitating direct visualization of the submucosal layer, resulting in efficient dissection due to the traction effect and adequate dissection depth. Use of this traction device can contribute to shortening the procedure time and reducing the risk of complications. This multicenter randomized controlled trial will evaluate the usefulness of the S-O clip in colorectal ESD and assess the procedure time and frequency of complications associated with the procedure.

**Methods/design:**

This multicenter, randomized control trial will enroll 200 patients at 4 hospitals in Japan undergoing ESD for colorectal epithelial tumors. Patients who meet the inclusion and exclusion criteria will be randomized to undergo ESD using S-O clips or conventional ESD. Patients will be randomized by a computer-generated random sequence with stratification by operator experience (trainee or expert), tumor location (colon/rectum), and institution. The primary endpoint will be ESD procedure time, defined as the time from the start of the local injection into the submucosal layer to the end of dissection. Other outcomes will include the rates of procedural complications, en bloc resection and cure.

**Discussion:**

ESD using the S-O clip is expected to shorten procedure time, reduce the incidence of adverse events, and standardize the procedure. This study may resolve clinical questions about whether ESD using the S-O clip traction device is more effective and safer than conventional ESD.

## 1. Introduction

Endoscopic submucosal dissection (ESD) is a technique that allows the en bloc resection of colorectal epithelial tumors regardless of size. Although ESD is minimally invasive and provides favorable outcomes, it is technically difficult to perform, due in part to a lack of maneuverability, and requires a long procedure time. In addition, patients undergoing colorectal ESD are at particularly high risk of complications due to the thin bowel wall, bowel flexion, and peristalsis, with prolonged colorectal ESD increasing the risk of complications such as perforation.^[1–3]^ Moreover, the colon is more susceptible than the esophagus and stomach to fibrosis caused by peristalsis and biopsy, especially in patients with recurrent lesions after EMR, with high fibrosis making the procedure more difficult. Patients may also experience postoperative fever and abdominal pain, even in the absence of obvious perforation, due to the transfer of heat during the procedure from the radiofrequency device to the muscle layer and serosa, a condition called post-ESD electrocoagulation syndrome (PECS). The incidence of PECS in patients undergoing gastric ESD has been reported to be 7.1%, but to be much higher, ranging from 12.1% to 40%, in patients undergoing colorectal ESD.^[4–7]^ Risk factors for PECS include long procedure time, lesion location other than in the rectum or sigmoid colon, tumor diameter >3 cm, and segmental resection.^[2,8,9]^ Therefore, the risk of PECS may be reduced by shortening the procedure time and minimizing the effect of thermocoagulation on the muscle layer.

**Figure 1.**
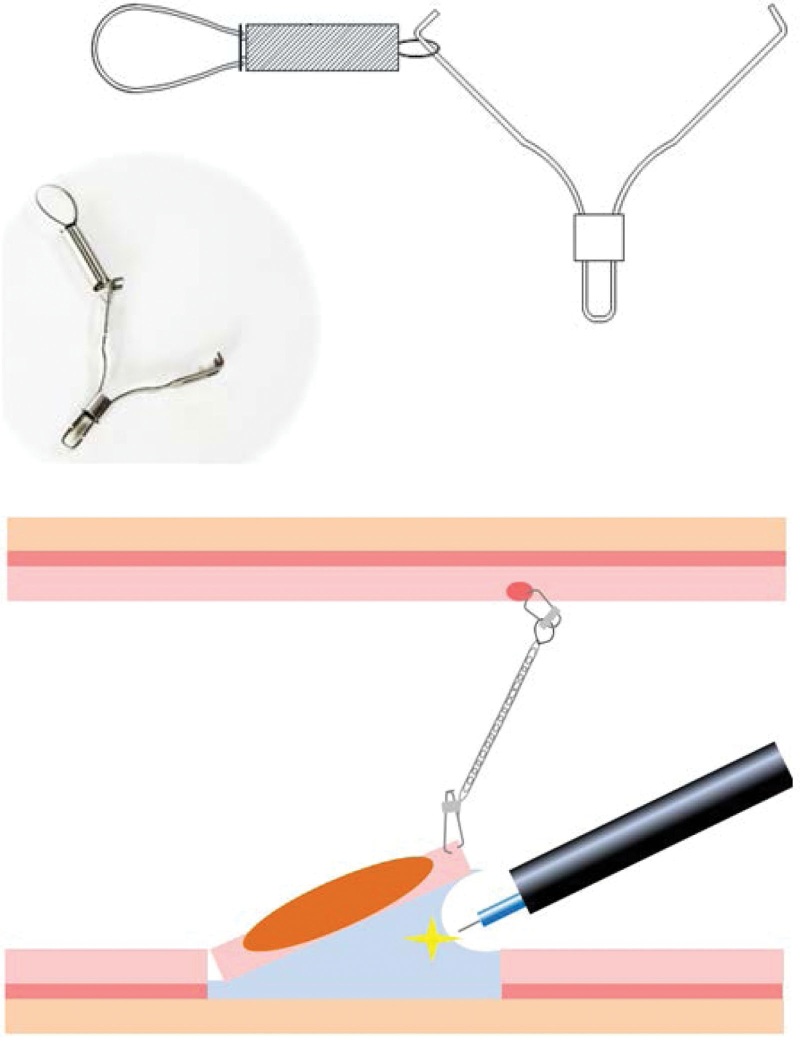
Photograph and schema of the S-O clip traction device. The spring effect provides continuous traction on the lesion, facilitating visualization of the avulsed surface.

Direct visualization of the submucosal layer, such as by traction of the lesion during mucosal dissection, may make ESD easier to perform. Several techniques involving traction of the lesion have been reported to be effective in ESD for early-stage colorectal cancer.^[10,11]^ One of these traction devices, the S-O clip (Zeon Medical, Tokyo, Japan), pulls the lesion in the intended direction in the lumen. The S-O clip can be passed through the working channel, allowing the lesion to be pulled independently of the endoscope. The S-O clip tractions the lesion toward the lumen and facilitates direct visualization of the submucosal layer, with the traction effect and adequate dissection depth allowing efficient dissection^[12]^ (Fig. 1). This traction device may shorten procedure times and reduce the risks of complications such as PECS. A single-center, randomized, controlled study performed at the institution that developed the S-O clip reported that use of the S-O clip in colorectal ESD significantly shortened the procedure time.^[8]^ To our knowledge, however, no multicenter randomized controlled trials have assessed the usefulness of the S-O clip in colorectal ESD. This clinical study was therefore designed to evaluate the usefulness of the S-O clip in colorectal ESD and assess the effects of this clip on procedure time and rate of complications.

## 2. Methods/design

### 2.1. Study aims and design

This prospective randomized clinical trial involving 4 medical institutions in Japan was designed to compare the efficacy and safety of the ESD procedure using the S-O clip with conventional ESD in patients with colorectal epithelial neoplasia.

### 2.2. Patients

At each institution, the on-site study investigators will enroll candidates who provide written informed consent, enter the required information into the electronic data collection system, verify that each candidate meets the eligibility criteria (i.e., the meets all inclusion criteria and none of the exclusion criteria), and register the candidate with the registration office. After confirming that the candidate meets the eligibility criteria, a registration number will be assigned. Once enrollment is complete, patients will be randomized 1:1 to undergo ESD using the S-O clip or conventional ESD. Patients will be stratified by 3 factors: ESD surgeon (trainee/expert), institution, and lesion site (rectum/colon).

### 2.3. Inclusion criteria

Patients will be included if they are aged ≥ 20 years, have an Eastern Cooperative Oncology Group Performance Status of 0 to 3, and have a colorectal epithelial tumor determined by colonoscopy to be an indication for ESD. Tumor size, morphology, circumferentially, and fibrosis are not required inclusion criteria. Lesions indicated for colorectal ESD will consist of those that require en bloc endoscopic resection and meet the 2014 colorectal ESD/EMR guidelines.^[14]^ These lesions will include the laterally spreading tumors-nongranular (LST-NG), lesions with a type VI pit pattern, T1 (SM) mildly invasive carcinomas difficult to resect en bloc by snare EMR, large depressed tumors, large raised lesions with suspected cancer, intramucosal tumors with fibrosis in the submucosa, sporadic localized tumors resulting from chronic inflammation, as in ulcerative colitis, and local residual early stage cancer after endoscopic resection. All patients must be fully informed and understand the requirements for study participation and provide voluntary written informed consent.

### 2.4. Exclusion criteria

Patients will be excluded if pre-operative examinations show indications of deep submucosal invasion; if the lesion is preoperatively diagnosed as a neuroendocrine tumor; if the tumor cannot be visualized in its entirety even with inversion; if the tumor crosses the anal canal, terminal ileum, appendix, or diverticulum; or if patients are eligible for ESD of ≥ 2 with colorectal tumors. Patients will also be excluded if their platelet counts are < 50,000/μL; if they are taking 2 or more antiplatelet agents or an anticoagulant other than warfarin; or if they are taking warfarin with a PT-INR ≥ 2.6, or a PT-INR ≥ 3.0 following mitral valve replacement surgery.^[13,14]^ Patients will also be excluded if they have serious complications in other organs, defined as an American Society of Anesthesiologists physical status ≥ 4; are pregnant; or are deemed ineligible for any other reason by the principal investigator or sub-investigator.

### 2.5. Randomization and blinding

Patients will be randomly assigned 1:1 to undergo ESD using the S-O clip or traditional study treatment groups using a minimization method. Randomization will be performed by dynamic balancing using the minimization method, with stratification by operator experience (trainee or expert), tumor location (colon/rectum), and institution. Neither patients nor physicians will be blinded to patient allocation. Endoscopists who have performed < 40 ESD procedures will be considered trainees, whereas those who have performed > 40 ESD procedures will be considered experts. Trainees who have performed < 40 colorectal ESD procedures are required to perform at least 5 of these procedures prior to the start of the study, with gastric ESD experience not required. In addition, each experts and trainee is required to perform at least 1 colorectal ESD with the S-O clip prior to the start of the study.

### 2.6. ESD procedures

The endoscope will be inserted into the colon; after identifying the lesion, its periphery will be marked when necessary. Solution will be injected into the submucosa and submucosal dissection will be continued until completion. There were no restrictions regarding endoscopes, needles, hemostats, and hoods, although only the Dual Knife (Olympus) was allowed for marginal incisions and submucosal dissection. Upper endoscopy was allowed, depending on the situation. EMR on other polyps was allowed before or after ESD on the same day. To ensure patient safety, for patients in whom it is impossible to complete the procedure, the trainee could be replaced by a specialist, thus allowing the procedure to continue. Trainees assigned to a procedure that has lasted more than 60 minutes but who are unable to continue the assigned procedure will be allowed to switch to the other procedure. Physicians having difficulty completing conventional ESD within 60 minutes will be allowed use of the S-O clip midway through the procedure. Conversely, physicians having difficulty completing ESD with the S-O clip within 60 minutes, due to interference by the S-O clip, will be allowed to remove the clip. Antithrombotic medications will be withdrawn or replaced according to gastrointestinal endoscopic practice guidelines.^[7,15]^

#### 2.6.1. Criteria for the ESD operators

All endoscopists performing ESD must be a principal investigator or sub-investigator; must have performed at least 5 colorectal ESD procedures; and must have performed at least 1 colorectal ESD procedure using the S-O clip.

#### 2.6.2. ESD procedure using the S-O clip

A procedural endoscope will be inserted into the colon. After local injection around the lesion, a mucosal incision will be made and submucosal dissection will be performed. If necessary, an additional local injection into the submucosal layer will be performed with the needle or high-frequency knife. After a circumferential incision, the S-O clip will be placed on the lesion. The ZEOCLIP will be used to pull the spring portion of the S-O clip and secure it to the contralateral wall. The number of clips used for traction is indeterminate. If the first S-O clip does not fit properly or detaches during the procedure, it may be reattached as appropriate. After applying traction, the lesion will be dissected and excised while visualizing the submucosal layer. Traction of the lesion is expected to facilitate visualization of the submucosal layer and allow complete resection.

#### 2.6.3. Conventional ESD procedure

A procedural endoscope will be inserted into the colon. After local injection around the lesion with a needle, mucosal incision and submucosal dissection will be repeated, with gravitational traction provided by a tip hood attached to the endoscope. Local injection into the submucosal layer with a needle or high-frequency knife will be performed when necessary. Submucosal dissection should excise the lesion without other traction devices.

### 2.7. Primary endpoint:

The primary endpoint of this study will be procedure time, defined as the interval from the start of local injection into the submucosal layer until the completion of dissection.

### 2.8. Secondary endpoints:

Several secondary endpoints will be evaluated. The first is the incidence of procedural complications (i.e., intraoperative perforation, delayed perforation, post-bleeding, PECS), defined as the ratio of the number of procedure-related complications to the total number of patients in the efficacy analysis population. PECS in the absence of obvious perforation will be defined as localized tenderness plus fever > 37.6°C, white blood cell count > 10.000/mm^3^, or C-reactive protein concentration ≥ 0.5 mg/dL within 6 hours after ESD. Posterior hemorrhage will be defined as a ≥ 2 g/dL decrease in Hb concentration or overt bleeding requiring endoscopic hemostasis, but not as a small amount of blood in the stool. Perforation will be defined as a total tissue defect that allows free passage to the body cavity, regardless of the presence of free air on radiographic examination.

Other secondary endpoints will include the *en bloc* resection rate, defined as the percentage of lesions resected en bloc without segmental resection; and the cure resection rate, defined as the percentage of resected specimens that meet all the following criteria on pathological evaluation: negative horizontal and vertical margins (complete resection); histological type such as papillary adenocarcinoma, ductal adenocarcinoma, or adenoma; evaluation of intramucosal carcinoma or submucosal invasion showing that the distance of submucosal invasion is < 1000 μm; and negative for lymphovascular and vascular invasion. Another secondary endpoint in patients who undergo ESD with the S-O clip will be the time from the start of traction with the S-O clip to the completion of detachment. An additional secondary endpoint will be the conversion rate, defined as the percentage of patients undergoing conventional ESD who required S-O clip placement during the procedure and the percentage of patients undergoing ESD with the S-O clip who required S-O clip removal. Finally, the incidence of adverse events will be evaluated.

### 2.9. Data collection

**Figure 2.**
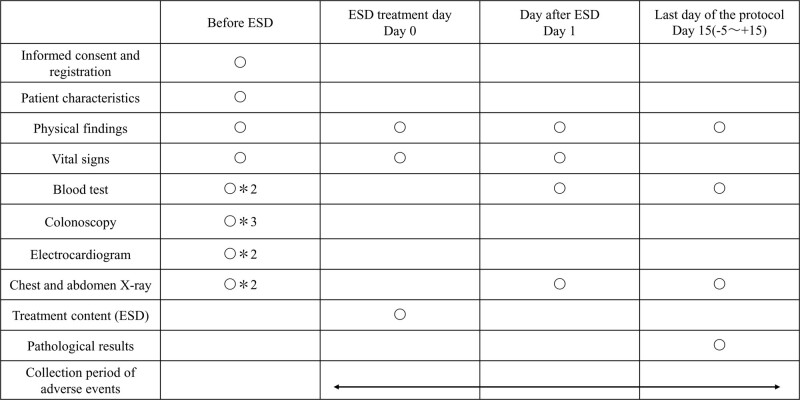
Schedule for data collection. *1: If adverse events associated with the procedure are suspected to have occurred, an emergency medical examination is performed as necessary. *2: within 28 days before registration. *3: within 120 days before registration.

Baseline assessment before ESD will include patient sex, date of birth, date of obtaining informed consent, history of gastrectomy or reconstructive surgery of the gastric tract, assessment of performance status, American Society of Anesthesiologists physical status, and current use of antithrombotic agents. Other assessments will include subjective findings; objective findings; and the results of hematologic examinations, blood coagulation tests, upper endoscopy, and X-ray examination of the chest. Treatment parameters will be assessed on the day of ESD, and adverse events (AEs) will be recorded from the day of ESD to discharge 15 days later (i.e., from −5 to + 15 days). The results of blood tests and X-ray examination of the chest will be evaluated the day after ESD. Parameters assessed at date of last evaluation will include physical findings and the results of blood tests, X-ray examinations, and pathological evaluations. The schedule for data collection is shown in Fig. 2.

### 2.10. Ethics approval and patient consent

The study protocol has been approved by the Ethics Committee of Wakayama Medical University (No. W-29), which confirmed that the protocol conformed to the tenets of the Declaration of Helsinki and Ethical Guidelines for Medical and Health Research involving Human Subjects. The trial has been registered with the Japan Registry of Clinical Trials (trial registration: jRCTs052190089). Written informed consent will be obtained from all patients enrolled in the study.

## 3. Statistical analysis

### 3.1. Sample size calculation

A single-center comparative study, in which 50 patients undergoing colorectal ESD were randomized, 27 to the S-O clip group and 23 to the conventional ESD group, found that the procedure time was significantly shorter in the S-O clip than in the conventional ESD group (37.4 ± 32.6 vs 67.1 ± 44.1 minutes, *P* = .03). Similarly, a previous study at Wakayama Medical University of patients who underwent treatment by a single surgeon found that the mean procedure time was shorter in the 57 patients who underwent colorectal ESD with the S-O clip (85.2 ± 52.3 minutes) than in the 42 patients who underwent conventional ESD (104.1 ± 60.3 minutes), although the difference was not statistically significant. Based on self-experimental data, the required number of cases was calculated to be 192 for exploration (significance level 0.1, power 0.8) and 324 for validation (significance level 0.05, power 0.9), with the required number of cases set at 200.

### 3.2. Statistical analysis

#### 3.2.1. Evaluation of the primary endpoint.

Multiple regression analysis will be performed using procedure time (i.e., from the start of localization to the completion of detachment) as response and allocation group and allocation factor as covariates. Point estimates, 95% confidence intervals, and *P* values for between-group differences will be calculated. In addition, summary statistics (mean, standard deviation, minimum, median, maximum) of treatment duration will be calculated for each group or ESD surgeon (trainee/expert).

#### 3.2.2. Evaluating secondary endpoints.

The complication rate will be defined as the rate of complications associated with the procedure. The frequencies and percentages of complications associated with the procedure, as well as the batch resection, cure, conversion, and adverse event rates will be tabulated for each group. Summary statistics (mean, standard deviation, minimum, median, maximum) from the time of S-O clip traction to the completion of dissection will be calculated for the S-O clip group.

### 3.3. Monitoring

The Monitoring Committee will collect information on patient enrollment, inclusion/exclusion criteria, and serious adverse events and provide feedback to participating sites on any problems for early resolution.

### 3.4. Adverse event reporting

An AE will be defined as any unfavorable or unintended injury or sign, including abnormal laboratory values, that occurs in any subject, regardless of whether or not it is causally related to the study and protocol treatment. Exacerbations of preexisting disease during the protocol treatment period will be regarded as AEs. Data on all AEs occurring between the start of treatment and the last observation specified in the protocol will be recorded. Serious AEs will be defined as deaths, life-threatening AEs, AEs requiring hospitalization or prolonged hospitalization for treatment, permanent or marked disability or dysfunction, or birth defects in offspring.

## 4. Discussion

ESD is a well-established technique that enables en bloc resection regardless of the size and morphology of the lesion.^[16–18]^ Although improvements in endoscopists’ skills and endoscopic equipment have increased the use of ESD for lesion removal, endoscopic manipulation remains difficult, with slight manipulations associated with a risk of complications. Perforation during colorectal ESD can cause peritonitis and require emergency surgery, emphasizing the importance of improving safety. Risks of perforations are higher when lesions are located vertically rather than tangentially, because, in the former, the tip of the knife approaches the submucosa perpendicularly. Safe and rapid dissection requires maintenance of an appropriate submucosal view that is as parallel to the muscle layer as possible. Thus, lesions require pulling in an effective direction. Conventional ESD uses a gravitational traction method that relies on changes in posture. Because this conventional method often cannot maintain sufficient traction, it often fails to maintain an appropriate submucosal view. This increases the difficulty of procedure increases, leading to an increased risk of complications. ESD using the S-O clip uses an endoscope and an independent device to maintain sufficient traction of the lesion in a short time regardless of lesion location. It has the advantage of allowing free manipulation of the scope.

This multicenter randomized controlled trial will evaluate the usefulness of the S-O clip in colorectal ESD by comparing the procedure time and frequency of complications with those obtained during conventional colorectal ESD. If use of the S-O clip shortens the procedure time and reduces the frequency of procedural complications, it may contribute not only to reducing the physical burden on patients, but also to shortening hospital stays and lowering costs. Although the number of facilities that can perform colorectal ESD is currently limited, safer performance of this procedure will allow it to become more widely performed. The major limitation of this trial will be that the operator will not blinded to treatment group allocation.

## 5. Conclusion

This prospective randomized clinical trial at 4 medical institutions has been designed to compare the efficacy and safety of colorectal ESD using the S-O clip with conventional ESD for colorectal epithelial neoplasia. ESD using the S-O clip may shorten procedure time, reduce the incidence of AEs, and standardize procedure. This study should resolve the clinical questions of whether ESD using the S-O clip is safer and more effective than conventional ESD for colorectal epithelial neoplasia.

## Author contributions

**Conceptualization:** Kazuhiro Fukatsu, Masayuki Kitano.

**Formal analysis:** Toshio Shimokawa.

**Funding acquisition:** Kazuhiro Fukatsu.

**Investigation:** Kazuhiro Fukatsu, Ikuharu Kinoshita, Ogata Syunsuke, Takao Maekita, Jun Kinoshita, Masaki Takao.

**Methodology:** Takao Maekita.

**Project administration:** Shinya Taki, Mikitaka Iguchi, Kazuhiro Fukatsu.

**Supervision:** Masayuki Kitano.

**Writing – original draft:** Shinya Taki.

**Writing – review & editing:** Shinya Taki, Mikitaka Iguchi, Masayuki Kitano.
